# Pedigree of Disease

**Published:** 1886-01

**Authors:** 


THE PEDIGREE OF DISEASE, *being Six Lectures on Temperament, Idiosyncrasy and Diathesis. Delivered in the Theatre of the Royal College, 1881. By* JONATHAN HUTCHINSON, F. R. S., *Professor of Surgery and Pathology in the College, etc. New York: William Wood & Co. 1885. Chicago.*

The point of view from which this old subject is treated is somewhat novel, and has received the approval of Sir James Paget in his Bradshaw Lecture of last year. Doubtless the recent progress of exact pathology has turned away attention, to a great extent, from such vague differences as those implied in the terms diathesis and temperament. Indeed, many pathologists, and especially those who follow the German investigators, practically ignore these terms as being too vague to be called scientific.

Older physicians used to prescribe for the patient's diathesis where we prescribe for his disease, and the minute classification of temperments, in which they delighted, only excite weariness when we recall them now. It is well to inquire, as the writer does however, whether this tendency has not gone too far, and see if it be true, as he claims, that we are now neglecting, unwisely, the study of those differences between man and man of which, for the most part, physiology takes no cognizance, but which may yet prove of much importance in modifying the processes of disease.

In these six lectures the subject is very clearly and comprehensively handled, so as to form a book full of suggestiveness to those who have had much clinical experience.

